# Supplementary material for: MetaRibo-Seq measures translation in microbiomes
Source: Nat Commun. 2020 Jun 29;11:3268. doi: 10.1038/s41467-020-17081-z (PMC7324362; doi:10.1038/s41467-020-17081-z)
Supplement: Supplementary file 10 — Supplementary Data 7 [file 41467_2020_17081_MOESM10_ESM.zip › File2/Confidence_High_Taxonomy/326638_out.krona.html]

Javascript must be enabled to view this page.

members
magnitude
magnitudeUnassigned
count
unassigned
taxon
rank

326638\_out

3

3
superkingdom
2

3
phylum
976

class
3
200643

3
order
171549

815
3
family

816
genus
3

species
3
310297

SRS049712\_contig\_number\_350SRS105082\_contig\_number\_1489SRS143876\_contig\_number\_2259
